# Inferring the Significance of the Polyamine Metabolism in the Phytopathogenic Bacteria *Pseudomonas syringae*: A Meta-Analysis Approach

**DOI:** 10.3389/fmicb.2022.893626

**Published:** 2022-05-06

**Authors:** Leandro Solmi, Hernán G. Rosli, Marina A. Pombo, Santiago Stalder, Franco R. Rossi, Fernando M. Romero, Oscar A. Ruiz, Andrés Gárriz

**Affiliations:** ^1^Laboratorio de Estrés Biótico y Abiótico en Plantas-Instituto Tecnológico de Chascomús (INTECh), Consejo Nacional de Investigaciones Científicas y Técnicas-Universidad Nacional de General San Martín (CONICET-UNSAM), Chascomús, Argentina; ^2^Laboratorio de Interacciones Planta Patógeno-Instituto de Fisiología Vegetal (INFIVE), Consejo Nacional de Investigaciones Científicas y Técnicas-Universidad Nacional de La Plata (CONICET-UNLP), La Plata, Argentina

**Keywords:** putrescine, spermidine, secondary metabolism, plant pathogen, *Pseudomonas syringae*

## Abstract

To succeed in plant invasion, phytopathogenic bacteria rely on virulence mechanisms to subvert plant immunity and create favorable conditions for growth. This process requires a precise regulation in the production of important proteins and metabolites. Among them, the family of compounds known as polyamines have attracted considerable attention as they are involved in important cellular processes, but it is not known yet how phytopathogenic bacteria regulate polyamine homeostasis in the plant environment. In the present study, we performed a meta-analysis of publicly available transcriptomic data from experiments conducted on bacteria to begin delving into this topic and better understand the regulation of polyamine metabolism and its links to pathogenicity. We focused our research on *Pseudomonas syringae*, an important phytopathogen that causes disease in many economically valuable plant species. Our analysis discovered that polyamine synthesis, as well as general gene expression activation and energy production are induced in the early stages of the disease. On the contrary, synthesis of these compounds is inhibited whereas its transport is upregulated later in the process, which correlates with the induction of virulence genes and the metabolism of nitrogen and carboxylic acids. We also found that activation of plant defense mechanisms affects bacterial polyamine synthesis to some extent, which could reduce bacterial cell fitness in the plant environment. Furthermore, data suggest that a proper bacterial response to oxidative conditions requires a decrease in polyamine production. The implications of these findings are discussed.

## Introduction

Polyamines comprehend a family of biological compounds that are required by all living organisms. At physiological pH, they are positively charged and bind to polyanionic compounds, such as RNA, DNA, proteins, and cell membranes (Michael, 2016). This ability explains their important participation in RNA translation, DNA replication, and membrane stability. The most abundant polyamines in bacteria are the diamine putrescine (Put) and the triamine spermidine (Spd) (Gevrekci, 2017), although polyamines with chemical similarities, such as cadaverine and norspermidine, might be also dominant in some species. In turn, the presence of the tetraamine spermine (which is abundant in mammals and plants) is rather rare.

**Figure 1:**
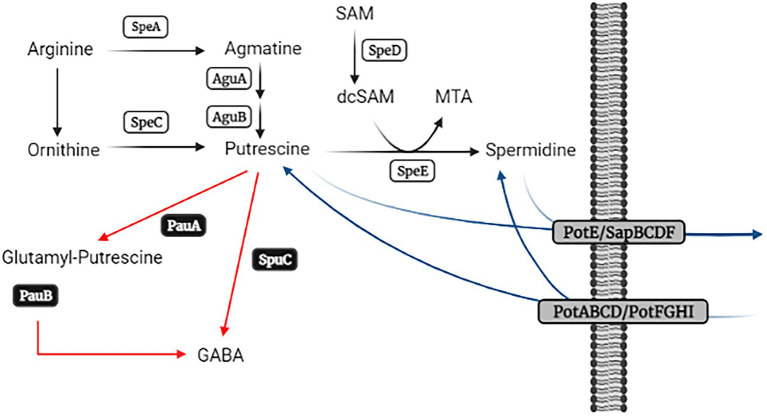
Schematic representation of polyamine metabolism pathways. Biosynthetic enzymes are represented in white rectangles, while black and grey rectangles depict the enzymes involved in polyamine catabolism and transport, respectively. Adapted from Schneider and Wendisch (2011) and created with BioRender.com.

The intracellular and extracellular contents of these compounds are tightly regulated by their synthesis, catabolism, and transport (Figure 1). Two pathways are used for the formation of Put, which depend on the initial substrate. One of them requires the decarboxylation of the amino acid arginine, whereas an alternative pathway decarboxylates ornithine instead. Decarboxylation of arginine is carried out by SpeA (arginine decarboxylase) to form agmatine, which is then converted into Put by two consecutive enzymatic steps catalyzed by agmatine deiminase (AguA) and *N*-carbamoylputrescine amidohydrolase (AguB). In turn, ornithine is decarboxylated by SpeC (ornithine decarboxylase) to yield Put as the end product. Then, SpeE (spermidine synthase) converts Put into Spd with the addition of amino propyl groups provided by decarboxylated S-adenosyl methionine, which is the product of the enzymatic step catalyzed by SpeD (S-adenosyl methionine decarboxylase). This anabolic pathway is highly conserved in bacteria, and numerous studies have demonstrated its importance in growth, stress tolerance, and virulence. (Shah and Swiatlo, 2008; Gevrekci, 2017; Igarashi and Kashiwagi, 2018; Chagneau et al., 2019; Banerji et al., 2020, 2021; Guerra et al., 2020; Nanduri and Swiatlo, 2021). Despite the fact that the catabolic and transport pathways have received less attention, there is evidence that they also make great contributions to polyamine homeostasis and cell fitness. (Johnson et al., 2012; Liu et al., 2018; Bolard et al., 2019). Several routes have been described for the degradation of polyamines in bacteria. Thus, they can first be converted into glutamyl derivatives by glutamyl-polyamine synthases and then oxidized to gamma-amino butyric acid (GABA) by glutamyl-polyamine oxidases. This involves the PauA and PauB protein families (as they are known in *Pseudomonas aeruginosa*), respectively (Schneider and Wendisch, 2011). Put might also be transaminated by SpuC using 2-oxo-glutaric acid or pyruvate as amino receptors to render gamma-amino butanal, which is then converted to GABA. Furthermore, the presence of a Spd dehydrogenase protein has been reported in some species (Hisano et al., 1992; Dasu et al., 2006), but this enzyme is not widely distributed in bacteria. In turn, a variety of transport systems participates in polyamine incorporation and secretion, and bacterial genomes generally exhibit redundancy in cognate genes (Schiller et al., 2000; Shah et al., 2008, 2011; Kurihara and Suzuki, 2015; Pipkins et al., 2017). Among these transporters, PotABCD, PotFGHI, PotE, and SapBCDF are the most studied. PotABCD and PotFGHI are involved in Spd and Put incorporation, respectively, whereas PotE and SapBCDF seem to be involved mostly in polyamine secretion.

The roles of these pathways in bacteria have mostly been studied in the model microbes *Escherichia coli* and *P. aeruginosa* (Wright et al., 1986; Xie et al., 1989; Lu et al., 2002; Chattopadhyay et al., 2003, 2009; Dasu et al., 2006; Chou et al., 2008; Kurihara et al., 2010; Yao et al., 2011; Wu et al., 2012; Kurihara and Suzuki, 2015; Sugiyama et al., 2016), and also in other human pathogens, such as *Streptococcus pneumoniae* (Banerji et al., 2021,^ 2022^; Nanduri and Swiatlo, 2021)*, Vibrio cholerae* (Karatan et al., 2005; Bridges and Bassler, 2021), and *Salmonella enterica* (Jelsbak et al., 2012; Guerra et al., 2020). We currently have a rather incomplete understanding of the importance of polyamines in the growth and virulence of phytopathogenic bacteria, even though some studies have revealed that they are essential in these organisms as well (Gerlin et al., 2021). For instance, it was reported that Spd is required for the synthesis of the toxin Phevamine A in *Pseudomonas syringae*, which helps to suppress the oxidative burst elicited in plant tissues following pathogen recognition (O’Neill et al., 2018). In addition, the secretion of Put by phytopathogenic bacteria has been observed in different bacterial species, such as *Ralstonia solanacearum*, *P. syringae*, and *Dickeya zeae* (Lowe‐Power et al., 2018; Vilas et al., 2018; Shi et al., 2019). Based on various reports demonstrating that polyamines can scavenge reactive oxygen species (ROS) and activate the antioxidant machinery (Bors et al., 1989; Das and Misra, 2004; Nayyar and Chander, 2004; Tang and Newton, 2005), it was proposed that Put secretion by bacteria constitutes a mechanism to counteract the oxidative stress imposed by plants at the site of the infection, a function that has yet to be confirmed. Alternatively, it has been postulated that Put secretion by *D. zeae* is engaged in cell-to-cell communication during the invasion of maize (Shi et al., 2019). More work is needed to reach a better understanding of the roles that polyamines play in such microorganisms.

As the number of molecular studies examining phytopathogenic bacteria is constantly growing, meta-analysis of publicly available transcriptomic data represents a valuable tool to comprehend the functionality of gene networks in pathogenesis (Caldas and Vinga, 2014). Thus, the comparison between these studies can lead to the recognition of common gene expression patterns and the identification of the metabolic pathways linked to cell behavior. Under this premise, we hypothesized that this kind of approach could enable us to discern the signatures characterizing the regulation of the metabolism of polyamines in bacterial pathogens of plants and discover potential connections between polyamine homeostasis and bacterial virulence.

We focused this study on transcriptomic data obtained from the bacterial species *Pseudomonas syringae*. This plant pathogen is worldwide distributed and more than 50 pathovars have been described, which altogether cause disease in the most important crops (Arnold and Preston, 2018; Xin et al., 2018). In addition, other plant and human pathogens were also considered to test whether identified expression signatures could be generalized to other pathogenic systems. Our study showed that polyamine production is first promoted during plant colonization but later suppressed, implying that these chemicals play an important role in the early stages of infection. In agreement with this, co-expression analysis revealed a close link between the expression of genes from the metabolism of polyamines and genes associated to essential cellular activities, such as gene expression regulation, energy production, transmembrane transport, and virulence. Interestingly, findings showed that the initial induction in synthesis are partially suppressed during the activation of plant defense responses, suggesting that plant immunity induction may affect bacterial polyamine homeostasis. In turn, even though the expression of genes from the catabolism and transport of polyamines varied among studies, we observed that polyamine incorporation is induced in later stages of infection. This probably helps to maintain the supply of polyamines when its synthesis is reduced. Another intriguing observation was that polyamine synthesis is repressed in response to oxidative stress, contrary to the widely held belief that polyamine production is essential for tolerance to these conditions. The implications of such observations are discussed.

## Materials and Methods

### Identification of Polyamine Metabolism Gene Orthologs in Bacterial Genomes

We followed two approaches to identify groups of polyamine metabolism gene orthologs in the different bacterial species considered in this work. In this trend, we explored the Ortholog Group Member database at the Pseudomonas Genome DB^1^, and besides, applied the reciprocal best hit method using the NCBI’s blastp software (Ward and Moreno-Hagelsieb, 2014). In those cases where these methods failed in returning potential orthologs, we still included in our analysis the best hits if the query coverage was higher than 80% and E-value was below 1 × 10^−10^. A list of the genes considered for each strain is shown in Supplementary Table S1.

### Selection of Transcriptomic Data Sets From the Public Domain

Microarray and RNAseq data were downloaded from the NCBI Gene Expression Omnibus (GEO). GEO repository was queried using keywords, such as “Pseudomonas,” “Phytopathogenic bacteria,” “pathogenic bacteria,” “oxidative stress,” and “plant infection.” In total, nine data sets were selected. Seven of these works were performed in three different pathovars of *P. syringae* as well as other species of the genus, such as *P. putida* and *P. aeruginosa*, whereas two considered the human pathogens *E. coli* and *S. enterica* (see Table 1 for a brief description). Studies in *P. syringae* growing under *in vitro* and *in planta* conditions were selected to explore the links between polyamine metabolism and pathogenesis, whereas those works evaluating bacterial responses to H_2_O_2_ were used to corroborate the potential role of these metabolites in the response to oxidative stress. In this last case, we excluded studies analyzing gene expression beyond 1 h of treatment, as we were interested in the early response. In addition, studies employing H_2_O_2_ concentrations greater than 15 mM were ruled-out since these concentrations are significantly higher than the physiological amounts encountered by bacteria in plant tissues. Importantly, because of the experimental heterogeneity associated to these data sets and the complexity underlying their normalization, we did not re-analyze them using a unified pipeline, but rather attempted to identify transcriptional patterns in a broader sense by comparing the expression levels reported by the authors. Thus, gene expression levels were obtained from the DEGs (Differentially Expressed Genes) listed in each study, and genes were included in our analysis if the fold change (log_2_FC) resulted >|0.5| and showed p-adjusted values <0.05.

**Table 1:** Description of the data sets used for the meta-analysis in this work.

Authors	Study accession	Bacterial strain	Plant host	Growth conditions
Nobori et al. (2018, 2020)	GSE103442 GSE138901	*P. syringae* pv. *tomato* DC3000	*Arabidopsis thaliana* Col-0	*In vitro* (minimal and rich media) and *In planta*
Lovelace et al. (2018)	GSE110100	*P. syringae* pv. *tomato* DC3000	*Arabidopsis thaliana* Col-0	*In planta*
Yu et al. (2013)	GSE42544	*P. syringae* pv. *syringae* B728a	*Phaseolus vulgaris* cultivar Bush Blue Lake 274	*In vitro* (minimal and rich media, stressful conditions) and *In planta*
McAtee et al. (2018)	PRJNA472664	*P. syringae* pv. *actinidiae* ICMP 18884	*Actinidia chinensis* Planch. var. *chinensis “Hort16A”*	*In planta*
Vandelle et al. (2021)	GSE164472	*P. syringae* pv. *actinidiae* biovars 1, 2 and 3	None	*In vitro* (minimal and rich media)
Bojanovič et al. (2017)	GSE85475	*P. putida* KT2440	None	*In vitro* (H_2_O_2_ amended media)
Jozefczuk et al. (2010)	GSE20305	*E. coli* MG1655	None	*In vitro* (H_2_O_2_ amended media)
Chang et al. (2005)	GSE3090	*P. aeruginosa* PA01	None	*In vitro* (H_2_O_2_ amended media)
Liu et al. (2020)	GSE 155479	*S. enterica* subs. *Enterica* serovar Enteritidis ATCC 13076	None	*In vitro* (H_2_O_2_ amended media)

Bacterial species/strains, plant host, and growth conditions are listed.

### Co-expression Analysis

Gene expression values (FPKM, fragments per kilobase of transcript per million mapped reads) uploaded by Nobori et al. (2018, 2020) and Yu et al. (2013) were used to perform a pair-wise Pearson correlation analysis across all the samples to generate a similarity matrix. This matrix was then used as input for the construction of co-expression networks (using R/WGCNA version 1.34; Langfelder and Horvath, 2008) as described by Sharma et al. (2018). Hierarchical cluster trees were created by setting gene co-expression module seizes of 30, deepSplit at level 1, and tree mergecutHeight at 0.20. Figures were created using Cytoscape (Killcoyne et al., 2009) and enriched GO terms were identified using BiNGO plugin (Maere et al., 2005).

## Results

### *P. syringae* Repress Polyamine Metabolism Under Apoplastic-Like Conditions

We looked for studies that compared the transcriptome of cells growing in hrp-inducing medium (HIM) with that of cells grown in rich media as a preliminary step in evaluating the link between polyamine homeostasis and bacterial virulence. HIM composition resembles nutritional conditions of plant apoplasts, such as low pH and reduced nitrogen/carbon ratios, and consequently induces the expression of the *hrp/hrc* genes. Importantly, the *hrp/hrc* cluster encodes the components of the type 3 secretion system (T3SS), a molecular structure that translocates protein effectors into plant cells and is essential for pathogenesis (Rico and Preston, 2008; Xie et al., 2019). In this trend, there were two works that met these requirements: Vandelle et al. (2021) employed a multistrain-whole genome platform to evaluate the responses in three biovars of *P. syringae* pv. *actinidiae* (*Psa1*, *2*, and *3*) as well as in *P. syringae* pv. *tomato* DC3000 (*Pst*), while Nobori et al. (2018) used RNAseq to investigate the transcriptional profile of *Pst*.

**Figure 2:**
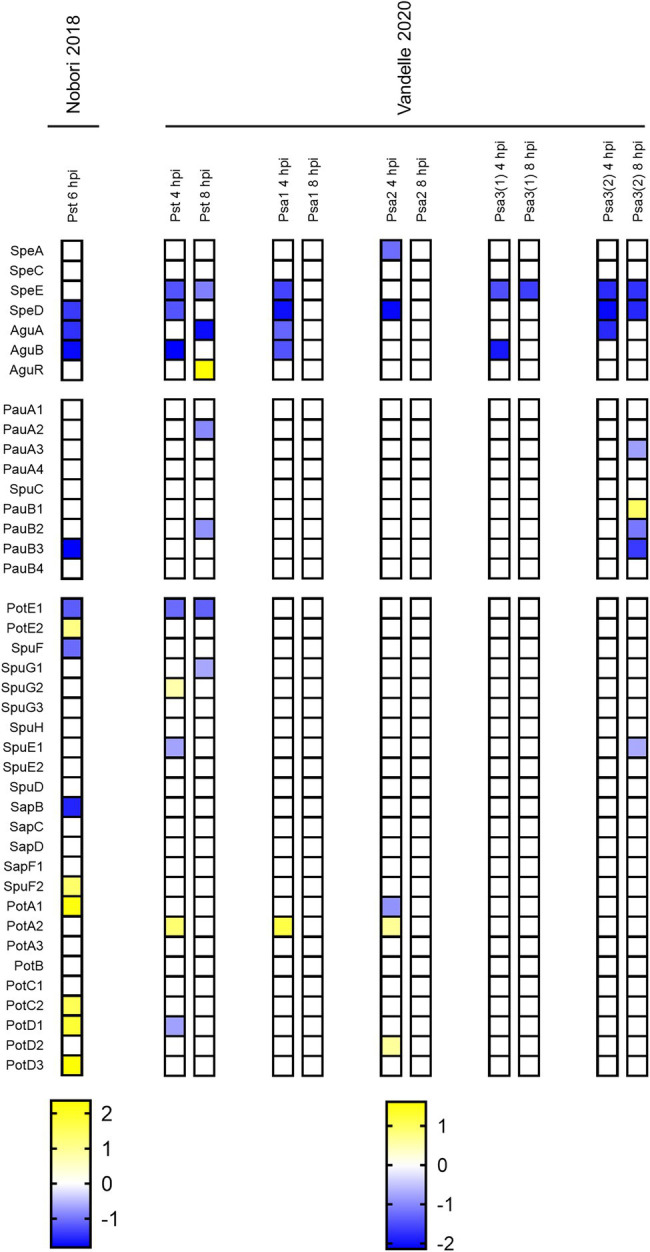
Heatmap representing polyamine metabolism gene expression levels in *P. syringae* during growth in apoplastic-like conditions compared to rich media. Color scales represent expression levels reported by the authors of indicated references. Gene expression levels were considered if log_2_FC > |0.5| and p adj < 0.05.

As shown in Figure 2, our analysis evidenced the downregulation of the anabolic pathway, even though the extent of the repression as well as the genes being repressed varied among both species and biovars. It’s worth noting that either *speE* or *speD* expression was reduced, implying that apoplast-mimicking conditions prevent Spd (but not Put) production. In turn, relatively minor changes were observed in the expression of genes involved in polyamine breakdown and transport. The most noticeable effects on these pathways were revealed in *Psa3(2)* as well as in *Pst*, which is consistent with the general gene expression similarities reported by Vandelle et al. (2021). Further research is needed to corroborate if the inhibition of polyamine metabolism under poor nutrient conditions is linked to the production of T3SS.

### Polyamine Metabolism Is Induced in *P. syringae* During Plant Invasion

We then expanded our investigation by analyzing transcriptomic profiles that were obtained from *Pseudomonas* species as they grew in plant tissues. Five studies using *Ps* pathovars were selected for this purpose: Lovelace et al. (2018) and Nobori et al. (2018, 2020) evaluated gene expression in *Pst* invading Arabidopsis, whereas Yu et al. (2013) and McAtee et al. (2018) focused their research in *P. syringae* pv. *syringae* B728a (*Pss*) and *Psa* colonizing *Phaseolus vulgaris* and kiwifruit, respectively.

**Figure 3:**
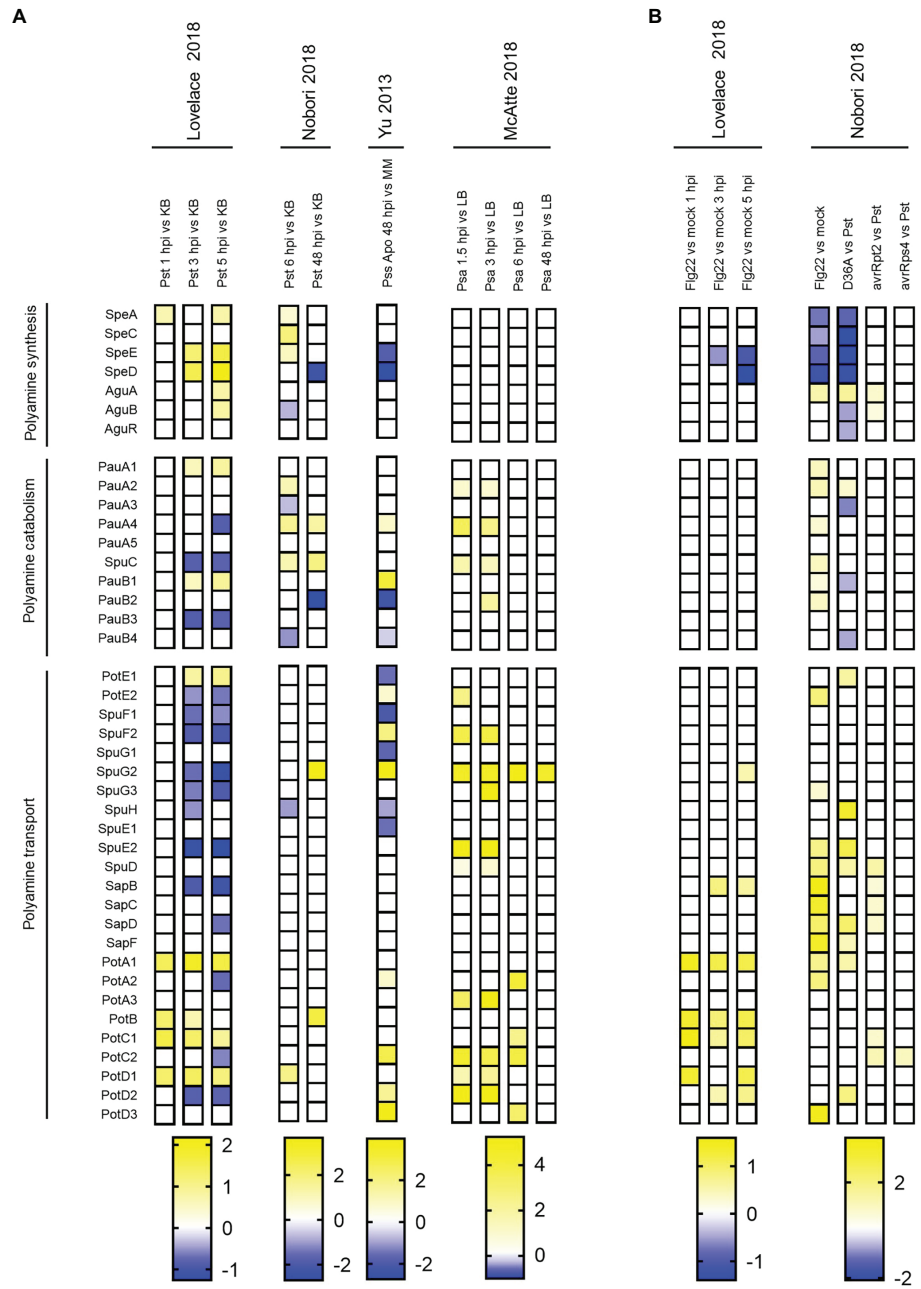
Polyamine metabolism gene expression in *P. syringae* during growth in plant tissues. Heatmap illustrating polyamine metabolism gene expression levels in *P. syringae* during **(A)** plant invasion (apoplastic growth) in comparison to basal conditions and **(B)** in flg22-treated plants compared to mock-inoculated plants (Flg22 vs. mock), and in non-virulent strains compared to WT *Pst* (D36A vs. Pst, avrRpt2 vs. Pst and avrRps4 vs. Pst). Color scales represent expression levels reported by the authors of indicated references, and they were considered if log_2_FC > |0.5| and p adj < 0.05.

In contrast to the expression profiles obtained during *in vitro* growth, this analysis shows that polyamine biosynthesis is induced early in *Pst* (within the first 6 h post-inoculation, 6 hpi; Figure 3A, first four columns). Thus, genes in charge of Put (*SpeA, SpeC, AguA*, and *AguB*) and Spd (*SpeD, SpeE*) synthesis were upregulated. In turn, no change was observed in the biosynthetic pathway of *Psa* during this stage of infection (see the first three columns corresponding to the data from McAtee et al., 2018). Genes conducing to the synthesis of Put were not differentially expressed later at 48 hpi, whereas the synthesis of Spd was suppressed at this stage in *Pst* and *Pss*. Therefore, we speculate that, at least for these strains, a rapid induction of polyamine production is important at the outset of plant invasion, and that this pathway is not required later with the development of the disease. The discrepancies between *Pst* and *Pss* with *Psa* suggest that the importance of the polyamine synthetic pathway may depend on the pathovar involved. In the interest of confirming if polyamine synthesis is significant for *Pss*, we searched for phenotypes in mutants of the cognate genes at the Fitness Browser database described at Helmann et al. (2019, 2020).^2^ In line with this, we found that the transposon-mediated disruption of the *speE* gene causes a minor negative fitness score when grown in the apoplast of green beans (data not shown), implying that the anabolism of Spd results relevant.

The examination of the expression of genes from polyamine catabolism and transport under *in planta* conditions, on the other hand, is rather inconclusive as many of them displayed contrasting expression patterns across the data set included. It is possible, then, that these pathways are more sensitive to different experimental setups and/or that their regulation is highly influenced by the bacterial genotype. In fact, while *Pst* and *Pss* show distinct regulatory effects on genes from the same pathway, almost all the genes differentially expressed in *Psa* are induced.

Two main defense responses are triggered in plants against the attack of potential pathogens (Nishad et al., 2020). The first layer of defense is activated upon the recognition of pathogen associated molecular patterns (PAMPs, such as the flagellin peptide flg22 and chitin) and is known as PTI (PAMP-triggered immunity). Even though pathogens introduce effectors into plant cells to counteract PTI and boost virulence (Macho, 2016), resistant plants can detect directly or indirectly some of these effectors initiating a second defense response that has been coined as Effector-triggered immunity (ETI) or nucleotide-binding Leu-rich repeat (NLR)-triggered immunity (NTI) (Lolle et al., 2020). Our analysis showed that pre-induction of PTI (by treating plants with flg22 before inoculation) hinders the upregulation of polyamine biosynthetic genes and causes the induction of genes from the transport systems SapBCDF and PotABCD (Figure 3B, flg22 vs. mock). In agreement with this, we also observed a relatively minor induction of polyamine biosynthesis in the effectorless polymutant *Pst*D36E (thus being unable to suppress plant immune system) compared to WT *Pst*, whereas components of the operon *sapBCDF/potABCD* were also induced. These findings suggest that plant defense mechanisms affect bacterial polyamine synthesis, resulting in the activation of polyamine incorporation to overcome this constrain. This also implies that the translocation of bacterial effectors helps to counteract the negative effects of plant defense responses on polyamine homeostasis.

In turn, the activation of ETI (evaluated through the inoculation with strains expressing either the avirulence genes *avrRPT2* or *avrRps4*) originated slight effects on bacterial polyamine metabolism, as only a higher induction of genes encoding polyamine transporters in the Pst-AvrRpt2 strain was verified. It should be considered though that ETI causes a rapid induction of plant defense mechanisms that strongly reduces pathogen’s growth. Thus, it is possible that the effects on bacterial polyamine metabolism could be masked on such drastic environmental conditions.

### Different Networks Are Coordinated With Polyamine Homeostasis in *P. syringae* During Plant Invasion

**Figure 4:**
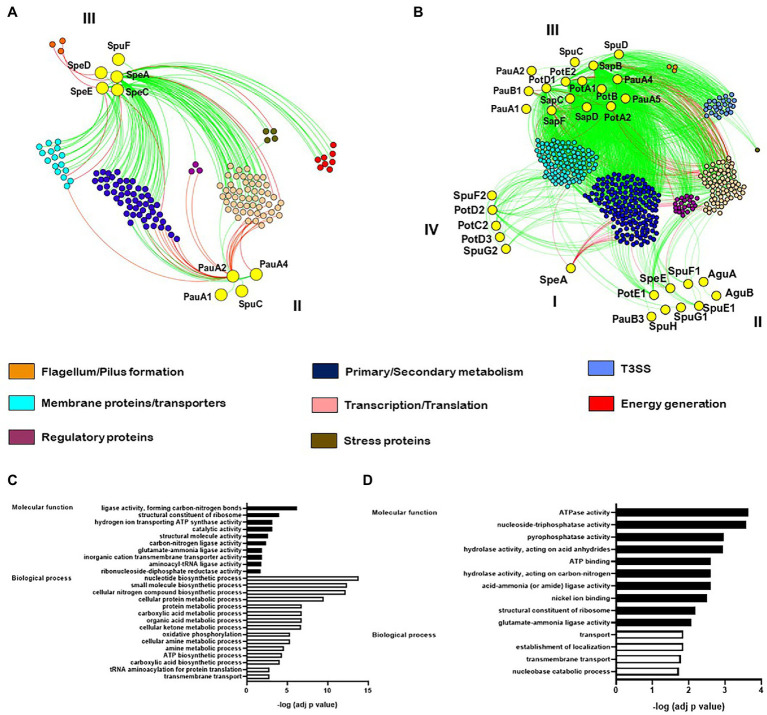
mRNA co-expression networks in *P. syringae* pv. tomato DC3000 using the data sets from Nobori et al. (2018, 2020) **(A)** and in *P. syringae* pv. *syringae* B728a from Yu et al. (2013) **(B)**. Red and green edges denote negative and positive correlations, respectively, for highly correlated genes (*R*2 > 0.75 was considered for Nobori et al., 2018, 2020; whereas *R*2 > 0.99 was considered for Yu et al., 2013). Functions are color-coded. Pearson’s correlation coefficients were used. Bar graphs in **(C, D)** depict GO enrichment analysis of the genes included in **(A, B)**, respectively.

We conducted a co-expression analysis with the goal of discovering gene modules coordinated with polyamine homeostasis. Our first analysis was made using the data collected by Nobori et al. (2018, 2020), that included numerous combinations of plant mutants and bacterial strains using the *Pst*/Arabidopsis model system. In this case, four genes coding for polyamine biosynthetic enzymes (*speA*, *speC*, *speE*, and *speD*), the polyamine transporter *potF*, and four involved in polyamine catabolism (*pauA1*, *pauA2*, *pauA4*, and *spuC*) were found to establish significant co-expression connections with other several gene nodes. An evaluation of these groups of genes as well as GO term enrichment analysis identified over-representation of gene categories intimately related to nitrogen compound biosynthesis, carboxylic acid metabolism, energy generation, primary metabolic processes, transmembrane transport, gene expression, and macromolecular biosynthetic processes (Figures 4A,C). Interestingly, we found that genes from different polyamine pathways established contrasting interactions with these nodes. Thus, whereas polyamine anabolic genes had positive co-expression scores with membrane transport, amino acid synthesis and regulation of gene transcription/translation, those involved in polyamine catabolism had negative scores, suggesting the existence of regulatory mechanisms that avoid simultaneous activation of both pathways. Another noteworthy observation is that polyamine biosynthesis had positive correlation with genes involved in energy generation (ATP synthase subunits and electron transport proteins) but negative correlation with genes involved in flagellum development (Figure 4A). Therefore, we conceive that energy production is closely linked to polyamine synthesis and that polyamines would not be required for bacterial movement activation (Figure 4A). To better understand the links in the regulation of these groups of genes we then conducted a dendrogram clustering on them. This analysis showed that the identified biosynthetic genes are grouped together in cluster III (Supplementary Figure S1A), which includes genes induced at the beginning of plant invasion (6 hpi) and repressed at later stages (48 hpi). Interestingly, these genes are also repressed in samples taken from PTI-induced plants or when the hypovirulent strain *Pst*-D36E was evaluated, as well as *in vitro* tests employing both rich and minimal media. In turn, the polyamine catabolic genes were assigned to cluster II. Even though this group is comprised by genes mildly induced in the early periods of plant invasion and repressed *in vitro*, they are strongly induced at later stages of the infection. Altogether, these findings support our earlier hypothesis that while polyamine production is essential at the start of plant invasion to maintain bacterial fitness, their accumulation is not required at later stages where the catabolic pathway is engaged to lower polyamine levels.

We applied the same analytical pipeline using the data set published by Yu et al. (2013), where gene expression was investigated in the strain *Pss* B728a during the infection of *P. vulgaris* 48 hpi (apoplastic and epiphytic growth), and also *in vitro* under different stressful conditions. After conducting a GO enrichment analysis, it was discovered that degradative processes and membrane transport categories were over-represented (Figure 4D). Besides, a substantially larger number of genes from polyamine metabolism were found exhibiting significant co-expression scores (Figure 4B), and strikingly, representatives from the distinct pathways were clustered together in different groups (Supplementary Figure S1B). Thus, it can be hypothesized that at this stage of infection (48 hpi), precise regulatory mechanisms affect specific genes from separate polyamine metabolic pathways rather than regulating an entire pathway. The biosynthetic gene *speA* is mostly linked by negative edges to genes from the secondary metabolism and regulators of gene transcription and translation (Figure 4B). This observation suggests that, as concluded above, polyamine synthesis may not be relevant for gene expression as it is required at the start of the process. In addition to that, *speA* was grouped in cluster I together with genes that are mildly repressed in response to low nitrogen availability but remained unaltered under other growing conditions (Supplementary Figure S1B). Thus, it is conceivable that bacteria face a reduction in nitrogen availability through the development of the disease, and consequently repress the expression of *speA* to limit the use of nitrogen in favor of the synthesis of other essential compounds. Cluster II contains the operon formed by the Put/Spd transport system PotFGHI, the Put exporter PotE(1), the catabolic oxidase PauB3, and the Spd synthase SpeE. These genes had positive correlations with gene expression regulators and transmembrane proteins slightly induced when cells are grown under osmotic conditions, suggesting that they play a role in the adaptation to this type of stress. In turn, cluster III includes the largest amount of polyamine metabolism genes. All these genes belong to the catabolic and transport pathways and establish positive correlation with a large amount of genes upregulated under low nitrogen conditions. It supports the notion that inducing polyamine degradation and transport aids in optimizing nitrogen usage under stressful conditions. Interestingly, these genes also showed positive edges with many genes that conform the *hrp/hrc* operons, indicating that their induction accompanies the activation of bacterial virulence. At last, cluster IV contains the polyamine transporter systems PotABCD/PotFGHI and other genes upregulated *in planta*, but not under any other condition. This observation would agree with the notion that transport of polyamines might play an important role during later periods of the infection.

### Polyamine Metabolism Is Shut-Down in Response to Oxidative Stress in *P. syringae*

Polyamines have been recognized as important metabolites that aid bacteria in their tolerance to oxidative stress (Chattopadhyay et al., 2003; Jung and Kim, 2003; Tkachenko et al., 2012; Akhova and Tkachenko, 2020; Felix et al., 2021). However, our co-expression analysis shown in the previous section did not evidence significant correlations with genes involved in redox homeostasis. The only exception is a positive correlation between the gene coding for the catalase KatG and the glutamyl-Put synthase PauA2 when the data from Nobori et al. (2018, 2020) were analyzed. As KatG (along with KatB) is required for full virulence in *Pst* (Guo et al., 2012) and its induction in response to H_2_O_2_ in *E. coli* is dependent on polyamines (Jung and Kim, 2003), we wondered if the activation of the detoxifying machinery in *P. syringae* growing under oxidative stress is accompanied by modifications in polyamine homeostasis. With this in mind, we began by searching for transcriptomic data studying bacterial gene expression in response to H_2_O_2_, focusing on works that stressed cells for a short period to assess the immediate response. As only Yu et al. (2013) evaluated this response in *P. syringae*, we included other two studies that used *P. putida* and *P. aeruginosa* to get a general sense of the behavior of the metabolism of polyamines in this bacterial genus. In addition to that, we also included other works in *E. coli* and *Salmonella enterica* to see whether there were any widespread regulatory mechanisms.

**Figure 5:**
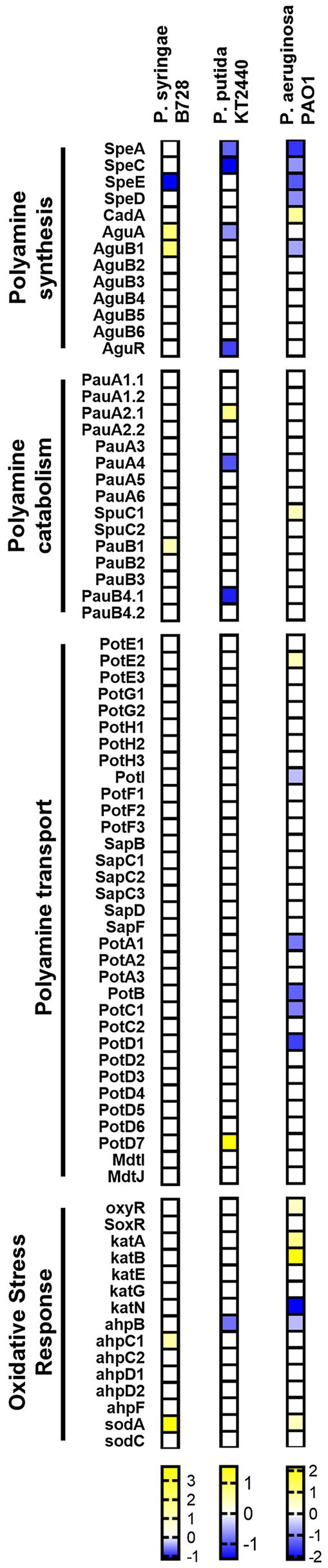
Heatmap showing polyamine metabolism and oxidative stress gene expression in *Pseudomonas* species during exposure to H_2_O_2_ in comparison to basal conditions. Color scales represent expression levels reported by the authors of indicated references. Gene expression levels were considered if log_2_FC > |0.5| and p adj < 0.05.

As shown in Figure 5, except for the inhibition of Put or Spd synthesis shortly after the addition of H_2_O_2_ to the culture media, our analysis shows that the metabolism of polyamines remains virtually unchanged in the response to oxidative stress in Pseudomonas species. A higher repression of polyamine synthesis and transport occurs in *E. coli* and *S. enterica* (Supplementary Figure S2), suggesting that the early regulation of the intracellular concentration of these metabolites might play a more important role in enterobacteria. In fact, known oxidative stress hallmark genes, such as *oxyR*, *soxS*, and *soxR*, resulted more highly activated in these species than in *Pseudomonads* at the time points analyzed. Overall, we conceive that a rapid repression of polyamine metabolism is required as part of the stress response in bacteria. It would be interesting to explore, then, how phytopathogenic bacteria adapt the metabolism of polyamines to cope with the oxidative stress imposed by plants without impacting cell fitness and pathogenesis.

## General Conclusion

The expanding amount of transcriptome data sets provides a valuable resource for identifying key features involved in cellular processes like growth, development, and stress tolerance (Caldas and Vinga, 2014). In the case of pathogenic microbes, these studies not only allow researchers to assess the relative relevance of different pathways but also provides a wealth of information about common mechanisms participating in the activation of virulence. In this work, we explored the importance of the metabolic routes maintaining polyamine homeostasis for disease and oxidative stress response in Pseudomonas species, with a focus on *P. syringae*.

We first compared polyamine metabolism gene expression levels in cells growing in minimal media in relation to those in rich media. Minimal media are thought to mimic apoplastic conditions leading to the induction of the virulence-associated *hrp/hrc* operons encoding for the T3SS (Rico and Preston, 2008). The studies selected for our analysis offered a time-lapse analysis spanning 8 h of culture and indicate the existence of a regulatory network suppressing polyamine homeostasis under minimal nutrient environments. These results curtail the importance of polyamines in supporting growth in low-nutrient media (meaning that more preferred metabolites would be synthesized instead under these circumstances) and besides, they also suggest that the mechanisms governing polyamine homeostasis are distinct from those inducing bacterial pathogenicity. Even though this observation contradicts our previous gene expression analysis in *Pst* using qRT-PCR (where the polyamine biosynthetic gene *speC* and the catabolic enzymes *pauA3* and *pauB2* resulted mildly upregulated in minimal medium), it should be considered that in the mentioned work only samples taken 6 hpi were analyzed and that variations in gene expression before or after that time could have been missed (Vilas et al., 2018).

An important point to be considered is that growth of the bacteria *in planta* generates much broader modifications of the bacterial transcriptomes, and consequently, results obtained from *in vitro* and *in planta* conditions are not always mirrored (Vandelle et al., 2021). Thus, we then evaluated then expression data from *in planta* studies since they should more accurately reflect the alterations of polyamine metabolism and its connection to disease. In line with this, our investigation discovered significant changes with respect to the metabolic behavior showed by cells *in vitro*, as the synthesis of polyamines was enhanced during the first stages of infection (up to 6 hpi) and repressed subsequently as pathogenesis progresses (48 hpi). If this initial rise in the contents of polyamines through their synthesis is required to support cell fitness at this stage, then mutant strains perturbed in polyamine synthesis should be affected in their virulence. Even though this kind of mutants has not been deeply tested in *P. syringae* yet, a genome-wide fitness profiling made on *Pss* by Helmann et al. (2019) showed that deletion of Δ*speE* conduces to mild negative fitness scores when growing in green pepper (Helmann et al., 2019). The fact that no other mutant strains affected in genes contributing to polyamine biosynthesis or transport demonstrated negative scores could be explained on the bases of pathway redundancy, which would assure the needed supply of polyamines when these pathways are partially affected. In relation to this, works in *Ralstonia solanacearum* (which exclusively depends on the SpeC pathway for Put synthesis) demonstrated that a Δ*speC* mutant is hypovirulent in tomato (Lowe‐Power et al., 2018).

Then there’s the question of why polyamines are needed for the first stages of plant infection. We could get a glimpse of their functions from our co-expression analysis based on the work made by Nobori et al. (2018, 2020), where most of the samples correspond to cells growing for 6 h both *in vitro* and *in planta*. This examination indicated that genes involved in polyamine biosynthesis were positively co-expressed with ribosome components as well as regulators of transcription/translation. On the contrary, polyamine catabolic genes showed a negative co-expression relationship with the same group of genes. This is in agreement with studies demonstrating that polyamines are required to assure correct ribosome ensemble and general transcription efficiency (Winther et al., 2021). We also distinguished the same pattern of co-expression with genes involved in ATP formation, carboxylic acid, and nitrogen metabolism. Thus, polyamine accumulation would be associated to the synthesis of important compounds and a higher rate of energy generation and gene expression.

The fact that polyamine biosynthetic genes were inversely linked with genes involved in flagella production is an interesting finding, since it suggests that bacterial movement could be affected by polyamines in *Pseudomonads*. In agreement with this, it was recently demonstrated that Put accumulation in *P. aeruginosa*, which is achieved by disrupting Spd synthesis or Put degradation, promotes cell transition toward the formation of sessile biofilms (Liu et al., 2021). However, this does not seem to be a general rule, as the link between polyamines and cell movement depends on the bacterial species. For instance, even though the lack of *speA* in *Dickeya zeae* and *Proteus mirabilis* decreases swimming and swarming mobilities (Shah and Swiatlo, 2008; Shi et al., 2019), deletion of *speA* and *speC* in *Yersinia pestis* perturbs biofilm formation (Patel et al., 2006).

We also evidenced that pre-induction of plant defense responses leads to a minor induction of polyamine biosynthesis and to the upregulation of polyamine transport genes. This also occurs when the effectorless D36E mutant strain is analyzed, emphasizing the importance of the T3SS function in avoiding the effects of plant defense mechanisms on pathogen’s metabolism. It is conceivable that the increased expression of polyamine transporters under these conditions could be an attempt to incorporate these compounds from the extracellular niche as a result of the reduced *de novo* production. But why is polyamine synthesis diminished after the elicitation of plant immunity? We hypothesize that the explanation to this phenomenon could be associated to 1) a bacterial response to the deleterious environment imposed by the activation of plant immunity, or 2) that plant defense responses directly target the synthesis of these amines to influence bacterial cell fitness. In connection with this, it is known that one of the main defense mechanisms deployed by plants against endophytic bacteria is the accumulation of reactive oxygen species at the apoplast (Yu et al., 2013; O'Leary et al., 2016), provoking oxidative stress conditions in the bacteria dwelling at this niche. With this is mind, we explored transcription data sets obtained from different bacterial species exposed to oxidative stress and found that, even though at different extents, polyamine biosynthetic genes were downregulated in bacteria. Hence, we speculated that the reduction in polyamine synthesis is more probable a consequence of a bacterial regulatory mechanism acting on polyamine synthesis, even though we cannot rule-out a possible direct effect of plant immunity on the expression of these genes. If lower expression of polyamine synthesis genes is associated with the activation of the antioxidative machinery, then it challenges the accepted paradigm that polyamines are required in bacteria for tolerance to oxidative stress (Chattopadhyay et al., 2003; Jelsbak et al., 2012; Johnson et al., 2012; El-Halfawy and Valvano, 2014). For instance, synthesis and secretion of polyamines by *R. solanacearum* at the tomato xylem was proposed by Lowe‐Power et al. (2018) as a mechanism that subdue this stress. Importantly, the authors demonstrated that Put is accumulated at this compartment presumably as a product of the activation of bacterial metabolism and that a Δ*speC* mutant resulted completely avirulent. In relation to this, we described in a previous work that *Pst* can also secrete large amounts of Put while growing in apoplastic washing fluids, although we could not find a link between this phenomenon and bacterial fitness/virulence. Our next experiments will try to generate *Pst* mutants unable to synthesize polyamines to further test their roles in stress tolerance and its link with pathogenesis.

As mentioned above, our study showed that bacterial polyamine biosynthesis is repressed at later stages of infection, which is also associated to the induction of polyamine transporters and catabolic genes. A similar scenario is observed in later stages of pathogenic interactions established by other bacterial species. For instance, *Xanthomonas axonopodis* pv. *glycines* reduces the expression of *speD* but upregulates other polyamine biosynthetic and transport genes after 72 hpi in soybean (Chatnaparat et al., 2016), whereas polyamine synthesis was repressed in *Xanthomonas oryzae* pv. *oryzae* after 5 days while polyamine transport was activated (Lee et al., 2017; data not shown). Importantly, our hierarchical clustering analysis of co-expressed genes shows that transcription profiles from samples taken 48 hpi are grouped with those obtained from cells growing *in vitro* under low nitrogen conditions or in minimal medium, as well as in experiments using primed plants (pre-infiltrated with flg22) or the *Pst* D36E strain. Thus, it is reasonable to conclude that bacteria are facing more restrictive conditions at the later phase of the plant invasion in terms of nutrient availability. Besides, the induction of a number of polyamine metabolic genes at 48 hpi is correlated with the induction of the T3SS. This observation agrees with the idea that the modulation of specific genes from the metabolism of polyamines, but not all, would be coordinated with the induction of bacterial virulence. More research is needed to fully comprehend the regulatory mechanisms governing polyamine metabolism throughout infection and their impact on pathogenesis.

**Figure 6:**
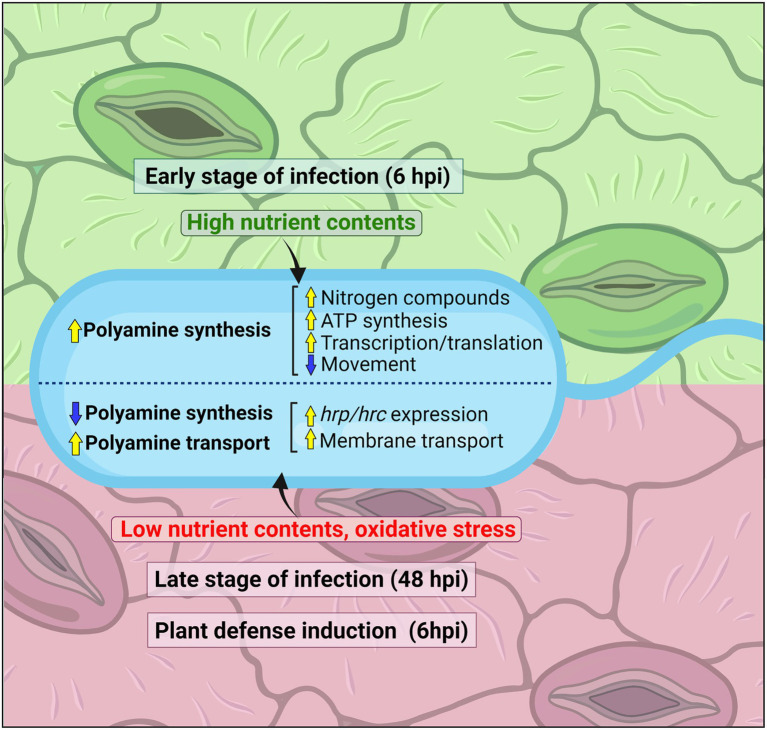
Scheme showing how the polyamine metabolism in *P. syringae* changes during plant invasion at different phases of infection. For the early stages of infection (6 hpi), polyamine metabolism regulatory hallmarks and their correlation with metabolic responses are mentioned on top of the figure, whereas for the late stages of infection (48 hpi) or the initial periods after induction of plant defensive responses are displayed below.

Altogether, these findings lead to the hypothesis that the synthesis of polyamines in phytopathogenic *Pseudomonas* species is important during the early periods of plant invasion, a process that is accompanied by the activation of nitrogen and carboxylic acid metabolism, general gene expression, and energy generation (Figure 6). This pathway, however, is repressed at the later stages of infection with the consequent induction of the polyamine transport and catabolic systems, a metabolic shift that would enable the survival of cells in the plant environment. Whether this behavior is extrapolated to other bacterial species should be evaluated with the generation of specific mutant strains perturbed in different branches of the polyamine metabolism.

## Data Availability Statement

The original contributions presented in the study are included in the article/Supplementary Material, further inquiries can be directed to the corresponding author.

## Author Contributions

LS and AG designed the study. SS, LS, and AG prepared figures. AG wrote the main text. All authors analyzed and reviewed the manuscript.

## Funding

LS, HR, MP, FR, FR, OR, and AG are members of the Research Staff of the Consejo Nacional de Investigaciones Científicas y Técnica*s* (CONICET, Argentina). This work was partly funded by the project “Universidades Agregando Valor” #8550 from the Secretaría de Políticas Universitarias, Argentina.

## Conflict of Interest

The authors declare that the research was conducted in the absence of any commercial or financial relationships that could be construed as a potential conflict of interest.

## Publisher’s Note

All claims expressed in this article are solely those of the authors and do not necessarily represent those of their affiliated organizations, or those of the publisher, the editors and the reviewers. Any product that may be evaluated in this article, or claim that may be made by its manufacturer, is not guaranteed or endorsed by the publisher.

## Footnotes

^1^ https://pseudomonas.com

^2^ https://fit.genomics.lbl.gov/cgi-bin/myFrontPage.cgi

## Supplementary Material

Supplementary Figure S1 Hierarchical clustering of highly correlated genes. Panels (A,B) correspond to the genes represented in (A,B), respectively.

Supplementary Figure S2 Polyamine metabolism and oxidative stress gene expression in *E. coli* and *S. enterica* in response to oxidative conditions compared to basal conditions. Color scales represent expression levels reported by the authors of indicated references. Gene expression levels were considered if log_2_FC > |0.5| and *p* adj < 0.05.

Supplementary Table S2 (A,B) described the samples included in Figures S1A,B as they are ordered in each panel from top to bottom, respectively.
