# Supplementary material for: Inferring the Significance of the Polyamine Metabolism in the Phytopathogenic Bacteria Pseudomonas syringae: A Meta-Analysis Approach
Source: Front Microbiol. 2022 May 6;13:893626. doi: 10.3389/fmicb.2022.893626 (PMC9120772; doi:10.3389/fmicb.2022.893626)
Supplement: Supplementary Figure S1 — Hierarchical clustering of highly correlated genes. Panels (A,B) correspond to the genes represented in (A,B), respectively. [file Data_Sheet_1.PDF]

A

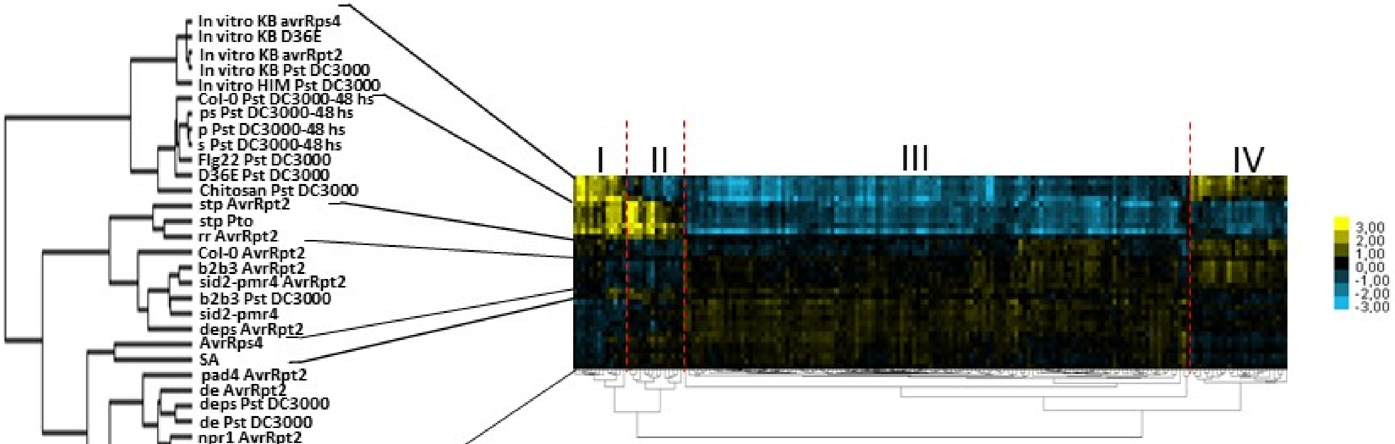

In vitro KB avrRps4
In vitro KB D36E
In vitro KB avrRpt2
In vitro KB Pst DC3000
In vitro HIM Pst DC3000
Col-0 Pst DC3000-48 hs
ps Pst DC3000-48 hs
p Pst DC3000-48 hs
s Pst DC3000-48 hs
Flg22 Pst DC3000
D36E Pst DC3000
Chitosan Pst DC3000
stp AvrRpt2
stp Pto
rr AvrRpt2
Col-0 AvrRpt2
b2b3 AvrRpt2
sid2-pmr4 AvrRpt2
b2b3 Pst DC3000
sid2-pmr4
deps AvrRpt2
AvrRps4
SA
pad4 AvrRpt2
de AvrRpt2
deps Pst DC3000
de Pst DC3000
npr1 AvrRpt2
npr1 Pst DC3000
ps AvrRpt2
sid2 AvrRpt2
pad4 Pst DC3000
ps Pst DC3000
sid2 Pst DC3000
mock
Col-0 Pst
I
II
III
IV
3,00
2,00
1,00
0,00
-1,00
-2,00
-3,00

B

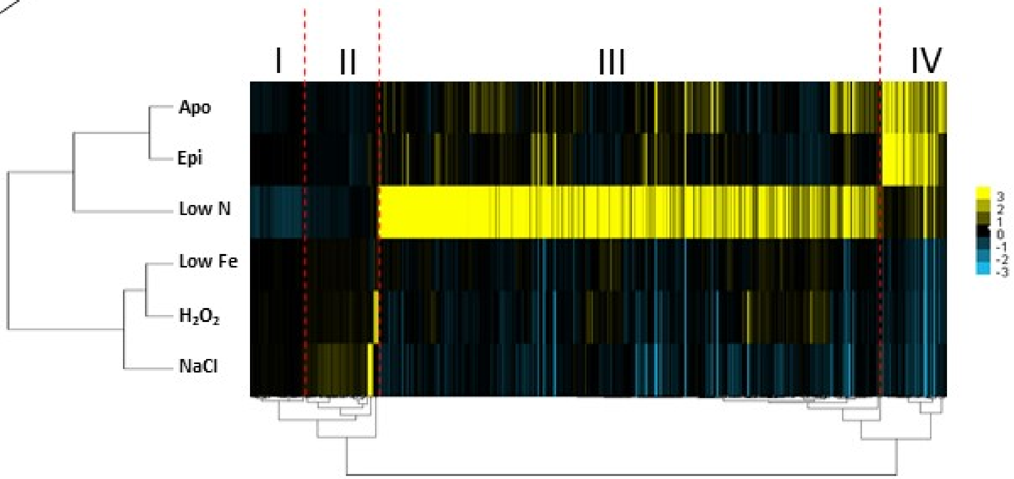

I
II
III
IV
Apo
Epi
Low N
Low Fe
H_2O_2
NaCl
3
2
1
0
-1
-2
-3

Table S2A

Sample name	Bacterial strain (phenotype)	Type of experiment	Culture media	Host (phenotype)	Time point
In vitro KB avrRps4	avrRpsa4 (ETI)	In vitro	KB	none	6 h
In vitro KB D36E	D36E (PTI)	In vitro	KB	none	6 h
In vitro KB avrRtp2	avrRpt2 (ETI)	In vitro	KB	none	6 h
In vitro KB Pst DC3000	Pst DC3000	In vitro	KB	none	6 h
In vitro HIM Pst DC3000	Pst DC3000	In vitro	HIM	none	6 h
Col-0 Pst DC3000-48 hs	Pst DC3000	In planta	none	Col-0	48 h
ps Pst DC3000-48 hs	Pst DC3000	In planta	none	Col-0 pad4 sid2 (affected in salicylic acid signalling)	48 h
p Pst DC3000-48 hs	Pst DC3000	In planta	none	Col-0 pad4 (affected in salicylic acid signalling)	48 h
s Pst DC3000-48 hs	Pst DC3000	In planta	none	Col-0 sid2 (affected in salicylic acid signalling)	48 h
Flg22 Pst DC3000	Pst DC3000	In planta-Flg22 pre-treated	none	Col-0	6 h
D36E Pst DC3000	D36E (PTI)	In planta	none	Col-0	6 h
Chitosan Pst DC3000	Pst DC3000	In planta-Chitosan pre-treated	none	Col-0	6 h
stp AvrRpt2	AvrRpt2 (ETI)	In planta	none	Col-0 stp1/stp13 (affected in sugar transport)	6 h
stp Pto	Pst DC3000	In planta	none	Col-0 stp1/stp13 (affected in sugar transport)	6 h
rr AvrRpt2	AvrRpt2 (ETI)	In planta	none	Col-0 rps2/rpm1 (affected in ETI triggering)	6 h
Col-0 AvrRpt2	AvrRpt2 (ETI)	In planta	none	Col-0	6 h

b2b3 AvrRpt2	AvrRpt2 (ETI)	In planta	none	Col-0 cyp79b2 cyp79b3 (affected in tryptophan metabolism)	6 h
sid2-pmr4 AvrRpt2	AvrRpt2 (ETI)	In planta	none	Col-0 spm (affected in salicylic acid signalling and callose deposition)	6 h
b2b3 Pst DC3000	Pst DC3000	In planta	none	Col-0 cyp79b2 cyp79b3 (affected in tryptophan metabolism)	6 h
sid2-pmr4	Pst DC3000	In planta	none	Col-0 spm (affected in salicylic acid signalling and callose deposition)	6 h
deps AvrRpt2	AvrRpt2 (ETI)	In planta	none	Col-0 dde2 ein2 pad4 sid2 (affected in salycilic, jasmonic acid and ethylen signalling)	6 h
pad4 AvrRpt2	AvrRpt2 (ETI)	In planta	none	Col-0 pad4 (affected in salicylic acid signalling)	6 h
de AvrRpt2	AvrRpt2 (ETI)	In planta	none	Col-0 dde2 ein2 (affected in salycilic, jasmonic acid and ethylen signalling))	6 h
deps Pst DC3000	Pst DC3000	In planta	none	Col-0 dde2 ein2 pad4 sid2 (affected in salycilic, jasmonic acid and ethylen signalling)	6 h
de Pst DC3000	Pst DC3000	In planta	none	Col-0 dde2 ein2 (affected in salycilic, jasmonic acid and ethylen signalling)	6 h
npr1 AvrRpt2	AvrRpt2 (ETI)	In planta	none	Col-0 npr1 (affected in salicylic acid signalling)	6 h

npr1 Pst DC3000	Pst DC3000	In planta	none	Col-0 npr1 (affected in salicylic acid signalling)	6 h
ps AvrRpt2	AvrRpt2 (ETI)	In planta	none	Col-0 pad4 sid2 (affected in salicylic acid signalling)	6 h
sid2 AvrRpt2	AvrRpt2 (ETI)	In planta	none	Col-0 sid2 (affected in salicylic acid signalling)	6 h
pad4 Pst DC3000	Pst DC3000	In planta	none	Col-0 pad4 (affected in salicylic acid signalling)	6 h
ps Pst DC3000	Pst DC3000	In planta	none	Col-0 pad4 sid2 (affected in salicylic acid signalling)	6 h
sid2 Pst DC3000	Pst DC3000	In planta	none	Col-0 sid2 (affected in salicylic acid signalling)	6 h
mock	Pst DC3000	In planta	none	Col-0 (control, mock-pretreatment)	6 h
Col-0 Pst	Pst DC3000	In planta	none	Col-0	6 h

Table S2B

Sample name	Bacterial strain (phenotype)	Type of experiment	Culture media	Host (phenotype)	Time point
Apo	Pss B728a	In planta	none	P. vulgaris	72 h
Epi	Pss B728a	In planta	none	P. vulgaris	48 h
Low N	Pss B728a	In vitro	Minimal médium lacking L-glutamine, $(NH4)_2SO_4$, and N-(β-ketocaproyl)-L-homoserine lactone	none	2 h
Low Fe	Pss B728a	In vitro	Minimal médium lacking $feCl_3$	none	2 h
H_2O_2	Pss B728a	In vitro	Minimal médium with 0.5 mM H_2O_2	none	15 min
NaCl	Pss B728a	In vitro	Minimal médium with 0.23M NaCl	none	15 min
