# Supplementary figures and images for: Inferring the Significance of the Polyamine Metabolism in the Phytopathogenic Bacteria Pseudomonas syringae: A Meta-Analysis Approach
Source: Front Microbiol. 2022 May 6;13:893626. doi: 10.3389/fmicb.2022.893626 (PMC9120772; doi:10.3389/fmicb.2022.893626)

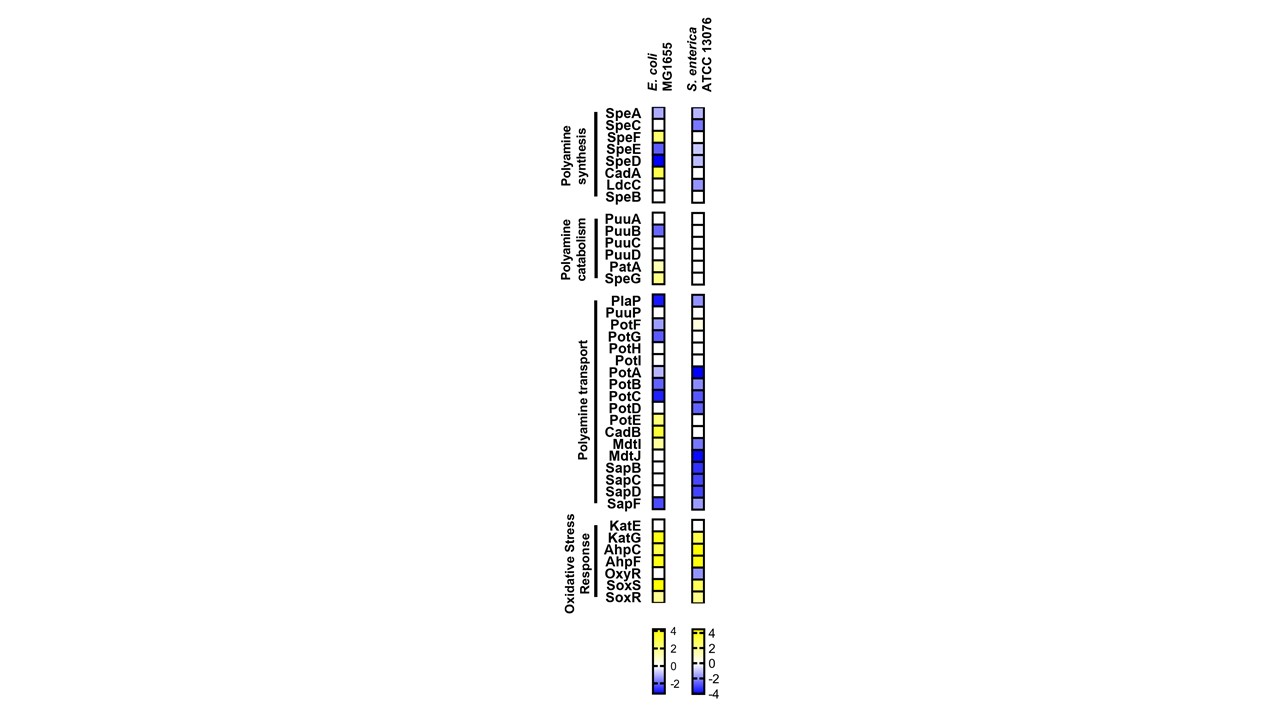

Supplement: Supplementary Figure S2 — Polyamine metabolism and oxidative stress gene expression in E. coli and S. enterica in response to oxidative conditions compared to basal conditions. Color scales represent expression levels reported by the authors of indicated references. Gene expression levels were considered if log2FC > |0.5| and p adj < 0.05. [file Image_2.jpeg]
