# Supplementary material for: Inferring the Significance of the Polyamine Metabolism in the Phytopathogenic Bacteria Pseudomonas syringae: A Meta-Analysis Approach
Source: Front Microbiol. 2022 May 6;13:893626. doi: 10.3389/fmicb.2022.893626 (PMC9120772; doi:10.3389/fmicb.2022.893626)
Supplement: Supplementary Table S2 — (A,B) described the samples included in Figures S1A,B as they are ordered in each panel from top to bottom, respectively. [file Table_1.docx]

**Table S1.** List of bacterial species and gene IDs considered in this work.

|  | **Gene name** | **P. syringae pv. tomato DC3000** | **P. syringae pv. actinidiae (ICMP 18884)** | **P. syringae pv. syringae B728A** | **P. aeruginosa PAO1** | **P. putida**  **KT2440** | **E. coli MG1655** | **S. enterica serovar enteritidis (ATCC 13076)** |
| --- | --- | --- | --- | --- | --- | --- | --- | --- |
| **Polyamine synthesis** | **SpeA** | PSPTO_4842 | IYO_RS04855 | Psyr_4381 | PA4839 | PP_0567 | b2938 | SEN2930 |
|  | **SpeC** | PSPTO_4572 | IYO_RS04125 | Psyr_4247 | PA4519 | PP_0864 | b2965 | SEN2958 |
|  | **SpeF** |  |  |  |  |  | b0693 | SEN0665 |
|  | **SpeE** | PSPTO_2055 | IYO_RS09100 | Psyr_1864 | PA1687 | PP_1867 | b0121 | SEN0171 |
|  | **SpeD** | PSPTO_0598 | IYO_RS02565 | Psyr_4575 | PA0654 |  | b0120 | SEN0170 |
|  | **SpeG** |  |  |  |  |  | b1584 | SEN1548 |
|  | **CadA** |  |  |  | PA1818 | PP_4140 | b4131/b0186 | SEN2539 |
|  | **SpeB** |  |  |  |  |  | b2937 | SEN2921 |
|  | **AguA** | PSPTO_5393 | IYO_RS28130 | Psyr_4932 | PA0292 | PP_0266 |  |  |
|  | **AguB** | PSPTO_5394 | IYO_RS28135 | Psyr_4933 | PA0293 | PP_0382  PP_4463  PP_0939  PP_3846  PP_0859  PP_0896 |  |  |
|  | **AguR** | PSPTO_5311 | IYO_RS27760 | Psyr_4869 | PA0294 | PP_0242 |  |  |
| **Polyamine catabolism** | **PauA1** | PSPTO_5310 | IYO_RS27825 | Psyr_4868 | PA0296 | PP_5184  PP_5299 | b1297 |  |
|  | **PauA2** | PSPTO_5309 | IYO_RS27820 | Psyr_4867 | PA0298 | PP_3184  PP_5183 |  |  |
|  | **PauA3** | PSPTO_1921 | IYO_RS09770 | Psyr_3492 | PA1566 | PP_4399 |  |  |
|  | **PauA4** | PSPTO_0359 | IYO_RS01995 | Psyr_2273 | PA2040 | PP_5046 |  |  |
|  | **PauA5** |  |  | Psyr_4817 | PA3356 |  |  |  |
|  | **PauA6** |  |  |  | PA5522 |  |  |  |
|  | **SpuC** | PSPTO_5308 | IYO_RS27815 | Psyr_4866 | PA0299 | PP_2180  PP_5182 | b3073 | SEN3060 |
|  | **PauB1** | PSPTO_2694 | IYO_RS14455 | Psyr_0158 | PA0534 | PP_2448 | b1301 | SEN2971 |
|  | **PauB2** | PSPTO_5074 | IYO_RS26025 | Psyr_0455 | PA1565 | PP_4893 |  |  |
|  | **PauB3** | PSPTO_0248 | IYO_RS01145 | Psyr_2427 | PA2776 |  |  |  |
|  | **PauB4** | PSPTO_0098 | IYO_RS00670 | Psyr_0232 | PA5309 | PP_5273  PP_3146 |  |  |
| **Polyamine transport** | **PlaP** |  |  |  |  |  | b2014 |  |
|  | **PuuP** |  |  |  |  |  | b1296 |  |
|  | **CadB** |  |  |  |  |  | b4132 | SEN2538 |
|  | **PotE** | PSPTO_5276  PSPTO_2026 | IYO_RS27035  IYO_RS10315  IYO_RS14305 | Psyr_4834  Psyr_1835  Psyr_2398 | PA2041  PA1819 | PP_5031 | b0692 | SEN0664 |
|  | **SpuF** | PSPTO_5303  PSPTO_2664 | IYO_RS27800 | Psyr_4863 | PA0302 | PP_5179 | b0855 | SEN0824 |
|  | **SpuG** | PSPTO_5302  PSPTO_2666  PSPTO_0488 | IYO_RS27795  IYO_RS14315  IYO_RS27265 | Psyr_4862  Psyr_2400  Psyr_4693 | PA0303 | PP_5178 | b0856 | SEN0825 |
|  | **SpuH** | PSPTO_5301 | IYO_RS27790 | Psyr_4861 | PA0304 | PP_5177 | b0857 | SEN0826 |
|  | **SpuE** | PSPTO_5306  PSPTO_0486 | IYO_RS27805  IYO_RS27810 | Psyr_4864  Psyr_4695 | PA0301 | PP_5180 | b0854 | SEN0823 |
|  | **SpuD** | PSPTO_5307 | IYO_RS27275 | Psyr_4865 | PA0300 | PP_5181 | b1293 |  |
|  | **SapB** | PSPTO_2575 | IYO_RS13730 | Psyr_2266 | - | PP_0881 | b1293 | SEN1340 |
|  | **SapC** | PSPTO_2574 | IYO_RS13725 | Psyr_2265 | - | PP_4455  PP_0880  PP_4454 | b1292 | SEN1339 |
|  | **SapD** | PSPTO_2573 | IYO_RS13720 | Psyr_2264 | - | PP_0879 | b1291 | SEN1338 |
|  | **SapF** | PSPTO_2572 | IYO_RS13715 | Psyr_2263 | - | PP_0878 | b1290 | SEN1337 |
|  | **PotA** | PSPTO_0562  PSPTO_3882  PSPTO_0489 | IYO_RS02370  IYO_RS20035  IYO_RS27260 | Psyr_4615  Psyr_1602  Psyr_4692 | PA3607 | PP_0411  PP_3817  PP_1484 | b1126 | SEN1823 |
|  | **PotB** | PSPTO_0564 | IYO_RS02380 | Psyr_4613 | PA3608 |  | b1125 | SEN1824 |
|  | **PotC** | PSPTO_0565  PSPTO_2665 | IYO_RS02385  IYO_RS14310 | Psyr_4612  Psyr_2399 | PA3609 | PP_3816  PP_1482 | b1124 | SEN1826 |
|  | **PotD** | PSPTO_0563  PSPTO_2785  PSPTO_2667 | IYO_RS02375  IYO_RS15285  IYO_RS14320 | Psyr_4614  Psyr_2516  Psyr_2401 | PA3610 | PP_0873  PP_3147  PP_3845  PP_5341  PP_3719  PP_2195  PP_1486 | b1123 | SEN1827 |
|  | **MdtI** |  |  |  | PA1540 | PP_1701 | b1599 | SEN1566 |
|  | **MdtJ** |  |  |  | PA1541 | PP_4930 | b1600 | SEN1567 |
